# TEAD inhibitors synergize with MEK, SHP2 and mTOR inhibitors in NF1 and NF2 cell lines

**DOI:** 10.17912/micropub.biology.002107

**Published:** 2026-04-26

**Authors:** Yang Yang, Sharavana Gurunathan, Shane Schechter, Jeffrey Field

**Affiliations:** 1 Systems Pharmacology and Translational Therapeutics, University of Pennsylvania, Philadelphia, PA, United States; 2 Harvard University, Cambridge, MA, United States

## Abstract

Neurofibromatosis type 1 (NF1) and *NF2* -related Schwannomatosis ( *NF2* -SWN) are both inherited syndromes characterized by Schwann cell tumors. NF1 tumors harbor activated Ras/MEK/ERK, while *NF2* -SWN tumors harbor activated mechanosignaling pathways, including Hippo/YAP-TAZ/TEAD. To test combinatorial strategies in tumor cell lines, we first screened a new-generation TEAD inhibitor, VT103, against 123 drugs and then validated the hits with pairwise titrations. VT103 consistently synergized with inhibitors of MEK (trametinib and selumetinib), SHP2 (TNO155) and mTOR (everolimus). The highest synergy ZIP score, calculated using SynergyFinder, was ~65 in the *NF2* -SWN cell line SC4, with lower magnitudes in an NF1 cell line.

**Figure 1. NF1 and NF2 -SWN screening and synergy analyses:** (A) Large-scale heatmap shows IC _50_ values for a compound panel in NF1 and *NF2* -SWN lines ± 2 µM VT103. (B) Focused heatmap highlights agents with VT103-induced IC _50_ reductions. (C) Bar plots ranking ZIP synergy scores from 6×6 matrices in ST88-14 (NF1), SC4 ( *NF2* -SWN), and HEI193 ( *NF2* -SWN), respectively.

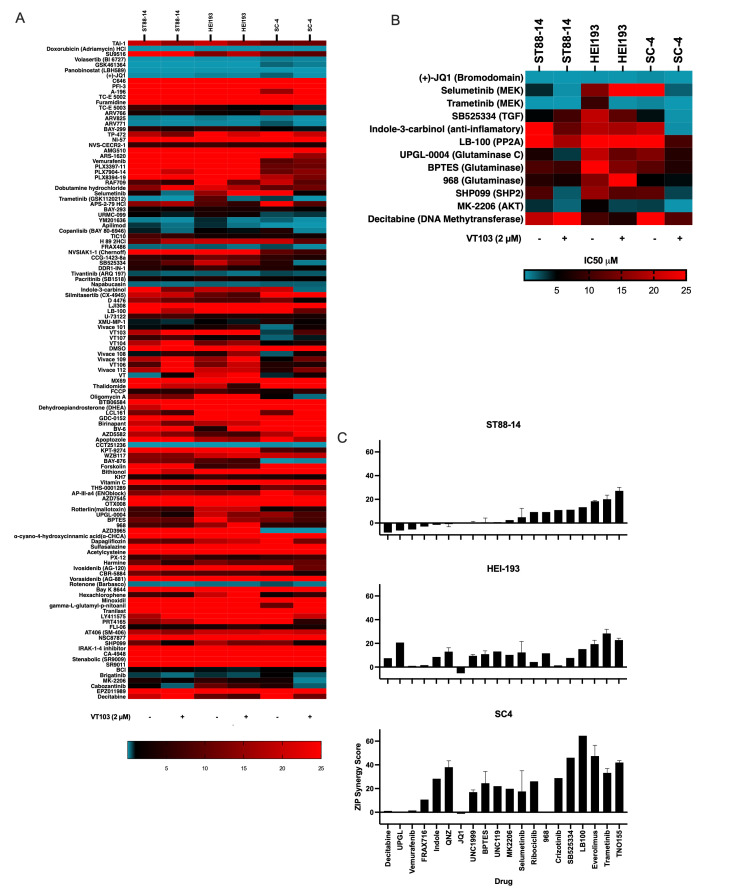



## Description

Neurofibromatosis type 1 (NF1) and type 2 (NF2) are both autosomal dominant syndromes driven by loss-of-function mutations in *NF1* (neurofibromin) and *NF2* (Merlin), respectively. Patients harbor mutant copies of either *NF1* or *NF2* , usually from birth. A major aspect of the morbidity of both syndromes is multiple benign tumors, which develop when sporadic mutations occur in the wild-type copy in Schwann cells. NF1 patients develop neurofibromas, a heterogeneous mixture of Schwann cells, mast cells and fibroblasts, while *NF2* -SWN patients develop Schwannomas, which primarily contain Schwann cells. About 10% of NF1 patients will develop malignant peripheral nerve sheath tumors (MPNST) from neurofibromas. NF1 and *NF2* -SWN cell comparisons are useful in drug screens because they are both derived from Schwann cells but have distinct primary driver mutations.

Neurofibromin is a RasGAP, so NF1 loss results in constitutive activation of RAS and its downstream signals through Raf/MEK/ERK and PI3K/mTOR. Several MEK inhibitors are FDA-approved to treat NF1 neurofibromas (Anastasaki et al., 2022). Though MPNST cell models respond to MEK inhibitors, MPNST patients show little response to MEK inhibitors as single agents or in combination with mTOR inhibitors (de Blank et al., 2022; Gross et al., 2020; Kim et al., 2026). More recently, the Hippo pathway was identified as an additional driver of MPNST (Wu et al., 2018).

Merlin is a cytoskeletal protein, and its loss in *NF2* -SWN tumors activates several mechanosignaling pathways through direct binding, including PAK/Rac and Hippo/YAP-TAZ/TEAD, but also interacts indirectly with Ras/MEK/ERK and mTOR signaling through mechanisms that are not well established. There are no FDA-approved treatments for *NF2* -SWN, though several drugs, including brigatinib (Plotkin Scott et al., 2024) and bevacizumab (Plotkin et al., 2019), have been beneficial in small-scale clinical trials (Evans, 1993).

The dependence of both tumors on Ras, mTOR and Hippo signaling suggests that combinations of drugs targeting these pathways may be beneficial for both NF1 and *NF2* -SWN tumors. It has been difficult to develop drugs against Hippo, but a new class of compounds that inhibit Hippo signaling by binding an auto-palmitoylation pocket in the YAP/TAZ partner, TEAD (Tang et al., 2021), shows some clinical efficacy in *NF2* -dependent mesotheliomas (Yap et al., 2025). There was also some efficacy in preclinical models of *NF2* -SWN either alone or together with PAK inhibitors (Benton et al., 2024; Laraba et al., 2023). We performed unbiased cell-based combination screens with a TEAD inhibitor, hypothesizing that, like YAP/TAZ knockdowns, it would synergize with inhibitors of Ras signals, such as MEK and mTOR inhibitors (White et al., 2019).

High-throughput screening (HTS) of drugs against cancer cell lines has been effective as a first step to prioritize drugs for animal clinical testing. The pioneering large-scale screening of a panel of 60 cell lines, the NCI-60, established that the most effective comparisons were between different cancer types, including those with different driver mutations (Shoemaker, 2006). HTS of drug pairs also helps prioritize drug combinations to test (Holbeck et al., 2017). We used a HTS strategy to screen NF1 and *NF2* -SWN cells. There are only a few NF1 and NF2 cell lines because they are rare tumors. The cell lines screened were the NF1 MPNST line ST88-14, the mouse *NF2* -SWN cell line SC4 and the human *NF2* -SWN Schwannoma cell line HEI193, which have been extensively characterized (Guo et al., 2017; Hung et al., 2002; Magallón-Lorenz et al., 2023; Teicher et al., 2015; Yang et al., 2011).

We screened 123 anti-neoplastic drugs using a library comprised of both classical chemotherapy and targeted therapeutics. The IC _50_ values were determined from eight concentrations of drugs ranging from 4.6 nM to 10 mM. Primary screening was done both with and without 2 mM of the TEAD1 selective inhibitor VT103 and summarized by a heatmap in Figure 1A (expanded in Figure 1B for clarity). VT103 was not active under these conditions, but it caused downward IC _50_ shifts for multiple compounds, suggesting it may potentiate their activity. To quantify hits and other candidate drugs, we next tested over 300 combinations of drugs and cells using 6×6 titrations in microtiter plates and then analyzed data with SynergyFinder (Zheng et al., 2022). In this algorithm, a zero-interaction potency (ZIP) synergy score >10 is highly synergistic. ZIP synergy scores >10 in at least one cell line were observed for eight of the 10 hits, with only decitabine and JQ1 yielding low ZIP synergy scores. The highest ZIP scores (~65) in SC4 were for VT103 combinations with MEK inhibitors (trametinib and selumetinib), SHP2 inhibitors (TNO155 was substituted for SHP099) (Ahmari et al., 2025; Wang et al., 2020), and mTOR inhibitors (everolimus). ST88-14 and HEI193 exhibited positive but lower synergy magnitudes, maintaining almost the same rank order of top VT103 partners. Comparable synergy scores were seen using three other TEAD inhibitors with several of these combinations. Weaker synergies were detected for glutaminase inhibitors (BPTES, UPGL, and 968) (Han et al., 2017) and the EZH2 inhibitor UNC1999.

VT103 synergized most consistently with inhibitors targeting the RAS/MAPK signaling (MEK, SHP2) and mTOR signaling in both NF1 and *NF2* -SWN cells. Data with PAK inhibitors suggest some synergy, but this was difficult to assess, as PAK inhibitors (Frax 486) are potent as single agents and strong single agents can reduce synergies in this assay (Benton et al., 2024; Guo et al., 2017). These findings suggest that VT103 deepens ERK pathway suppression. This may be because loss of YAP/TAZ activation causes an activation of ERK. Thus, inhibiting ERK is needed after Hippo inhibition. The strongest synergy was observed in the *NF2* -SWN line SC4.

The Hippo pathway effectors YAP/TAZ drive *NF2* -SWN tumors as well as Ras-driven tumors that become resistant to MEK inhibitors (Edwards et al., 2023; Kim et al., 2016; Lin et al., 2015; White et al., 2019). YAP and TAZ are partners for TEAD, which combine to make a functional transcription factor. While most studies to date have tested the Hippo pathway using genetic methods to inhibit YAP and TAZ, which cannot yet be inhibited with small molecules, it remains unclear whether inhibition at the level of TEAD is sufficient. Our data suggest TEAD inhibitors have partial effects as single agents, but they are strongly synergistic with Ras pathway inhibitors. Surprisingly, despite multiple TEAD isoforms, tests with multiple TEAD inhibitors suggest that inhibition of TEAD1 alone may be sufficient to inhibit *NF1* and *NF2* -SWN cells. The synergy with glutaminase and epigenetic inhibitors suggests metabolic and epigenetic pathways may also be exploitable with TEAD inhibitor combination strategies.

**Limitations of this study:** Cell screening studies can identify combinations as starting points for animal and perhaps clinical studies. However, only 5-10% of cell and animal studies translate to clinical benefit. For example, MEK and MTOR inhibitors (Kahen et al., 2018), which are cytostatic in MPNST animal models, shrink tumors in combination, but were of little benefit in a clinical trial (Kim et al., 2026).

## Methods

ST88-14 identity was confirmed by STR profiling. Cell lines were grown and treated using a small molecule panel of inhibitors using a high-throughput format, as described, in the absence and presence of 2 µM VT103 (Guo et al., 2017). About 1000 cells per well were plated, and after ~24 hours, they were treated with drugs, incubated for ~72 hours and then tested for viability using ATPlite/luciferase analysis. The IC _50_ values were computed from dose–response curves, and compounds showing VT103-induced IC _50_ reductions were advanced into 6×6 dose-matrix combination assays. Drug concentrations for the matrices varied using 0x, 0.25x, 0.5x, 1x, 2x, and 4x of each drug, where x is the IC _50_ from panel A. Scoring used the ZIP reference model in SynergyFinder with default settings (Zheng et al., 2022).

**Data sharing.** Primary data from this manuscript are shared on Synapse, and an interactive heat map showing dose-response titrations of the drugs tested in Panel A is available on the Pharmacomb website (https://www.med.upenn.edu/fieldlab/). Raw and extended data for this manuscript is available on the Synapse portal: Field, J. (2026). NF Cell Line Compound Screens. Synapse. https://doi.org/10.7303/SYN11817821
